# Global Perspective on Acute Kidney Injury in Uganda

**DOI:** 10.34067/KID.0000000611

**Published:** 2024-10-08

**Authors:** Grace Kansiime, Dan Oriba Langoya, Robert Kalyesubula

**Affiliations:** 1 Department of Internal Medicine, Mbarara University of Science and Technology, Mbarara, Uganda; 2 Department of Internal Medicine, St Mary's Hospital Lacor, Gulu, Uganda; 3 Department of Physiology and Department of Internal Medicine, Makerere University College of Health Sciences, Kampala, Uganda; 4 Nephrology Section, Yale School of Medicine, New Haven, Connecticut

**Keywords:** AKI, health equity, diversity, and inclusion, hemodialysis access, supportive care

## Introduction

AKI represents a significant health challenge particularly in low- and middle-income countries such as Uganda.

Managing AKI in Uganda presents numerous challenges, largely influenced by the country's socioeconomic context, health care infrastructure, and resource limitations. Uganda is among Africa's fast-growing countries with a population of over 48 million in 2023, yet resource-poor with 42% of its population living on less than USD 2 per day.^1^ This demographic transition, coupled with few health care professionals trained in the management of AKI, limits the capacity for early recognition, appropriate management, and timely referral of patients, leading to suboptimal outcomes.

In this article, we explore the burden of AKI, peculiar risks and predispositions, its management, and dialysis financing in Uganda.

## Burden of AKI in Sub-Saharan Africa and Uganda

### Incidence and Prevalence

The burden of AKI in sub-Saharan Africa is mostly unknown because of the diverse nature of studies on the subject. The incidence ranges from 5.7% to 33.3% among patients admitted to general wards to >50% among those in the intensive care units (ICUs).^2,3^ In Uganda, AKI is the most common kidney disease among children, with a prevalence of 13.5% among hospitalized children and up to 43.5% among children with cerebral malaria.^4^ In adults, the prevalence is more varied and it is about 9% among those receiving contrast (contrast-associated AKI) for various procedures, 16.3% among adults with sepsis and HIV,^5^ and 34.4%–46.9% among those with severe burns or trauma.^6^

### Causes

The causes of AKI in Uganda are varied along the life spectrum. Peculiar causes of community-acquired AKI are shown below (Table 1). AKI is mostly caused through a common pathway of pre-renal AKI secondary to dehydration. Malaria and diarrheal diseases are the most common causes among children as Uganda has the third highest global burden of malaria. AKI has been shown to prolong hospital stay and increase in-hospital and postdischarge mortality and long-term neurocognitive impairment among children with malaria.^4^ Among adults, causes include sepsis, tuberculosis, HIV, environmental exposures, and toxins.^2,5,7^ Uganda has experienced recurrent outbreaks of viral hemorrhagic fevers over the past 20 years, including Ebola, Marburg, Congo Crimean, and the most recent Rift Valley fever outbreak in September 2023.^8^ Many patients with viral hemorrhagic fever present to the hospital with severe AKI as a complication, for example, 26.7% of patients with Ebola present with AKI,^9^ which is thought to be due to renal hypoperfusion, inflammatory response, and interstitial nephritis secondary to viral injury. Obstetric complications, including hypertensive diseases of pregnancy, puerperal sepsis, and hemorrhages, still contribute to AKI. AKI incidence is up to 42.8% among women with severe preeclampsia.^10^

**Table 1:** Common causes of community-acquired AKI in Uganda

Pre-Renal Causes	Renal/Parenchymal Causes	Postrenal/Obstructive Causes
Infections Malaria especially in children TB HIV Diarrheal diseases Acute hemorrhagic fevers, rift valley fever Obstetric causes Preeclampsia and HELLP syndrome Septic abortions Puerperal sepsis Severe hemorrhage; APH and PPH Surgical causes Trauma Burns	ATN mostly because of uncorrected pre-renal AKI Infections Malaria VHFs Environmental exposures and toxins Herbalism including local herbs, herbal teas Snake bites Toxins like herbicides and pesticides, detergents Common medications associated with AKI Anti-TB drugs: rifampicin, isoniazid, nonsteroidal anti-inflammatory drugs like diclofenac, antibiotics: gentamycin; vancomycin Contrast-associated AKI Acute GN	Benign Benign prostatic hyperplasia Malignant Prostate cancer

APH, antepartum hemorrhage; ATN, acute tubular necrosis; HELLP, hemolysis, elevated liver enzymes, low platelets; PPH, postartum hemorrhage; TB, tuberculosis; VHF, viral hemorrhagic fever.

### Mortality Associated with AKI in Uganda

AKI has consistently been associated with high mortality. The mortality risk among children with AKI is about 3.5 times higher compared with those without AKI, even postdischarge.^4^ Mortality is high among patients with AKI because of severe burns or trauma,^6^ sepsis or tuberculosis (up to 21%),^2,5^ and up to 52.5% among those with AKI in the ICU.^3^

### Diagnosis of AKI

In Uganda, community-acquired AKI primarily because of dehydration and hypotension frequently presents in advanced stages. To enhance early detection, high-risk hospitalized patients are regularly assessed on the basis of risk factors, like advanced age, comorbidities, and exposure to nephrotoxic drugs. This includes consistent monitoring of kidney function, urine output, and administering renal-dosed medications.

Serum creatinine testing is the main diagnostic method for AKI, yet its availability is often limited, particularly in lower-tier health facilities, and where available, patients may bear the costs themselves. Although urinalysis tests are more accessible, comprehensive urine microscopy is rarely performed because of a lack of resources, including microscopes and trained personnel. Further diagnostic procedures, such as complete blood counts and microbiology cultures, are generally restricted to referral centers. Advanced diagnostic tools, like kidney biopsies and newer biomarkers, remain largely within research contexts rather than routine clinical practice.

### Management of AKI

The management of AKI in Uganda is primarily supportive, focusing on identifying and treating underlying causes, such as sepsis with dehydration and hypotension in adults and malaria and dehydration in children. This includes addressing complications, adjusting medications, and managing fluid balance. However, drug stock-outs in public health facilities often force patients to purchase necessary medications and supplies out of pocket.

General doctors and physicians typically manage initial AKI cases, whereas patients needing KRT are referred to nephrologists. This underscores the challenges in accessing comprehensive care, especially for specialized treatments.

## Dialysis Services for AKI in Uganda

### Availability of Dialysis for AKI

Uganda has made strides in expanding dialysis centers over the past 5 years, but access to dialysis and ICU services remains abysmal.^7^

**Figure 1:**
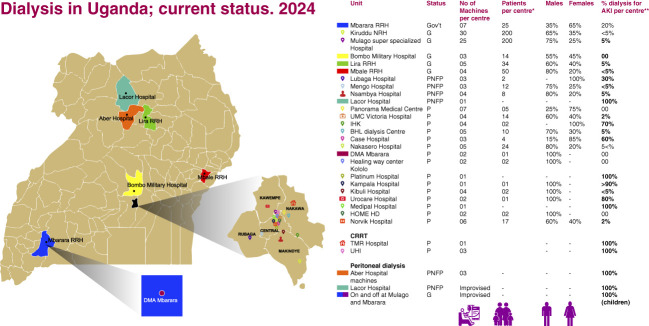
**Dialysis centers in Uganda 2024.** *Total number of patients on chronic dialysis per center. **Percentage number of patients on dialysis for AKI per center. BHL, building healthy life; CRRT, continuous renal replacement therapy; DMA, DMA Clinic and Diagnostics Ltd; Gov't/G, Government Center; HD, hemodialysis; IHK, International Hospital Kampala; NRH, National Referral Hospital; P, private center; PNFP, private not for profit; RRH, Regional Referral Hospital; UHI, Uganda Heart Institute Ltd; UMC, unihealth medical centre. Illustration by Richard Mugisha, Uganda.

Only six of 135 districts have dialysis centers, each district serving populations of 400,000 to 1.5 million. In addition, 75% of these centers are concentrated within a 10-km radius of Uganda's capital, Kampala (Figure 1).

High costs and limited availability hinder access to life-saving dialysis for most patients. Those who can afford treatment may need to travel over 100 km to a center. Patients with AKI, including women with pregnancy-related AKI and children with malaria, account for about 10% of those on hemodialysis. Peritoneal dialysis is occasionally used for AKI, often with locally made fluids because of resource constraints. As of August 2024, Uganda had only three continuous KRT machines, all located in private hospitals.

## Costs

In Uganda, hemodialysis is an out-of-pocket treatment, costing between $20 and $45 per session in public hospitals—equivalent to 10 days' wages for the average Ugandan—and $90–$150 in private units or about 24 days' wages. Continuous KRT costs approximately $2000 per session (24–72 hours), whereas peritoneal dialysis costs $30–$50 per session. Despite government subsidies for dialysis in public hospitals, it remains unaffordable for most. Occasionally, fees are waived for impoverished patients for their first five sessions, but this requires complicated administrative approvals, making the process lengthy and cumbersome.

## Challenges

The lack of universal health insurance and high out-of-pocket costs for medications and dialysis severely affect patients with AKI. With only 14 nephrologists, 84 dialysis nurses, and ten dialysis technicians for a population of 48 million, the health care workforce is critically insufficient. In addition, limited access to ICUs for severe AKI cases compounds these challenges, underscoring the urgent need for systemic health care reforms.

## Recommendations

1. Preventive measures: Understanding the precise epidemiology of AKI in Uganda is essential for prioritizing prevention among at-risk populations. Health workers should receive regular continuous medical educations and continuous professional developments focused on AKI prevention, early diagnosis, and management, especially at lower health facilities, to improve patient outcomes.
2. Infrastructure development: Uganda needs to invest in infrastructure to expand dialysis services and improve ICU availability. Each regional hospital should at least have trained staff and dialysis and ICU services for AKI management.
3. Financial support: The Ugandan Government and international organizations should provide financial support to make dialysis more accessible and affordable, particularly for the most vulnerable with AKI (children and women).

## Supplementary Material
