# Supplementary material for: Global Perspective on Acute Kidney Injury in Uganda
Source: Kidney360. 2024 Oct 8;6(1):152–5. doi: 10.34067/KID.0000000611 (PMC11793190; doi:10.34067/KID.0000000611)
Supplement: Supplementary file 1 [file kidney360-6-152-s001.pdf]

ASN Journal Disclosure Form

As per ASN journal policy, I have disclosed any financial relationships or commitments I have held in the past 36 months as included below. I have listed my Current Employer below to indicate there is a relationship requiring disclosure. If no relationship exists, my Current Employer is not listed.

R. Kalyesubula reports the following:
Employer: Makerere University

I understand that the information above will be published within the journal article, if accepted, and that failure to comply and/or to accurately and completely report the potential financial conflicts of interest could lead to the following: 1) Prior to publication, article rejection, or 2) Post-publication, sanctions ranging from, but not limited to, issuing a correction, reporting the inaccurate information to the authors' institution, banning authors from submitting work to ASN journals for varying lengths of time, and/or retraction of the published work.

Name: Robert Kalyesubula
Manuscript ID: K360-2024-000433R1
Manuscript Title: Global Perspective on AKI in Uganda," submitted to the Kidney360
Date of Completion: September 27, 2024
Disclosure Updated Date: September 27, 2024

ASN American Society of Nephrology
JASN Journal of the American Society of Nephrology
KIDNEY360 Accessing Our World From Every Angle
CJASN Clinical Journal of the American Society of Nephrology

ASN Journal Disclosure Form

As per ASN journal policy, I have disclosed any financial relationships or commitments I have held in the past 36 months as included below. I have listed my Current Employer below to indicate there is a relationship requiring disclosure. If no relationship exists, my Current Employer is not listed.

G. Kansiime reports the following:
Employer: Mbarara University of Science and Technology

I understand that the information above will be published within the journal article, if accepted, and that failure to comply and/or to accurately and completely report the potential financial conflicts of interest could lead to the following: 1) Prior to publication, article rejection, or 2) Post-publication, sanctions ranging from, but not limited to, issuing a correction, reporting the inaccurate information to the authors' institution, banning authors from submitting work to ASN journals for varying lengths of time, and/or retraction of the published work.

Name: Grace Kansiime
Manuscript ID: K360-2024-000433R1
Manuscript Title: Global Perspective on AKI in Uganda
Date of Completion: September 27, 2024
Disclosure Updated Date: August 20, 2024

ASN
American Society of Nephrology
JASN
Journal of the American Society of Nephrology
KIDNEY360
Accessing Our World From Every Angle
CJASN
Clinical Journal of the American Society of Nephrology

ASN Journal Disclosure Form

As per ASN journal policy, I have disclosed any financial relationships or commitments I have held in the past 36 months as included below. I have listed my Current Employer below to indicate there is a relationship requiring disclosure. If no relationship exists, my Current Employer is not listed.

O. Langoya reports the following:
Employer: St. Mary's Hospital Lacor, Gulu Uganda

I understand that the information above will be published within the journal article, if accepted, and that failure to comply and/or to accurately and completely report the potential financial conflicts of interest could lead to the following: 1) Prior to publication, article rejection, or 2) Post-publication, sanctions ranging from, but not limited to, issuing a correction, reporting the inaccurate information to the authors' institution, banning authors from submitting work to ASN journals for varying lengths of time, and/or retraction of the published work.

Name: Oriba Dan Langoya
Manuscript ID: K360-2024-000433R1
Manuscript Title: Global Perspective on AKI in Uganda
Date of Completion: October 1, 2024
Disclosure Updated Date: October 1, 2024
